# Drinking water salinity and blood pressure in coastal Bangladesh: follow-up of a population-based cohort in a climate-vulnerable region

**DOI:** 10.1136/bmjopen-2026-116453

**Published:** 2026-07-01

**Authors:** Aneire E Khan, Eurydice Costopoulos, Sontosh Kumar Mojumder, Asma Begum Shilpi, Mohammad A Hoque, Adrian Butler, Christopher Millett, Naila Z Khan, Kristine Belesova, Paolo Vineis

**Affiliations:** 1 Department of Epidemiology and Biostatistics, School of Public Health, Imperial College London, London, England, UK; 2 Upazila Health Complex Dacope, Khulna, Bangladesh; 3 Clinical Neurosciences Center, Bangladesh Protibondhi Foundation, Dhaka, Bangladesh; 4 School of the Environment and Life Sciences, University of Portsmouth, Portsmouth, UK; 5 Department of Civil and Environmental Engineering, Imperial College London, London, UK; 6 Public Health Policy Evaluation Unit, School of Public Health, Imperial College London, London, UK; 7 Public Health Research Centre and Comprehensive Health Research Center (CHRC), National School of Public Health, Lisbon, Portugal; 8 Department of Epidemiology and Biostatistics, School of Public Health, Imperial College London, London, UK

**Keywords:** Epidemiology, Public health, Cardiovascular Disease, Hypertension

## Abstract

**Objectives:**

To investigate the association between drinking-water sodium concentration and blood pressure among non-pregnant women living in a high-salinity, climate-vulnerable coastal region of Bangladesh.

**Design:**

Follow-up study of women originally enrolled in a population-based study conducted during pregnancy between 2009 and 2011.

**Setting:**

Rural coastal subdistrict in southwest Bangladesh affected by saltwater intrusion and seasonal drinking-water insecurity.

**Participants:**

Of 1208 women enrolled in the original study, 900 were located in 2018 and 740 non-pregnant women were re-enrolled and included in the present analysis. Eligible participants were women from the original study who were alive and still residing in the study area; women who had migrated could not be located, declined participation or were pregnant at follow-up were excluded.

**Primary and secondary outcome measures:**

The primary outcome was blood pressure, measured using a standardised protocol. Main exposure was sodium concentration in household drinking water and reported drinking-water source. Secondary analyses assessed temporal changes in drinking-water sodium between 2009 and 2018 and associations with water source, perceived taste and changes in exposure over time.

**Results:**

Mean sodium concentration in household drinking water was highest in groundwater/tubewell users (933 mg/L, SD 372). Approximately 22% of women consumed drinking water contributing up to 94% of the WHO-recommended maximum daily sodium intake, assuming 2 L/day consumption. Sodium concentrations increased in seven of nine administrative unions between 2009 and 2018 (p=0.013). Compared with women consuming water containing <200 mg/L sodium, exposure to >1000 mg/L was associated with higher odds of raised diastolic blood pressure (adjusted OR 2.30, 95% CI 1.40 to 3.77; p=0.001). Restricted cubic spline and piecewise regression models suggested a non-linear association, with a threshold near 1000 mg/L. No statistically significant association was observed for systolic blood pressure.

**Conclusions:**

High sodium concentrations in drinking water were associated with elevated diastolic blood pressure among women in coastal Bangladesh. Drinking-water salinity may represent an under-recognised cardiovascular risk in climate-vulnerable coastal populations. These findings support the need for salinity surveillance, climate-resilient low-sodium drinking-water supplies and further research to inform health-based standards for sodium in drinking water.

**Trial registration number:**

Not applicable.

**STRENGTHS AND LIMITATIONS OF THIS STUDY**

- Drinking-water sodium exposure was assessed using objective household and source water sampling, alongside a questionnaire capturing season-specific water-source use (dry season, rainy season and time of survey).
- Blood pressure was measured using a standardised protocol with ≥3 readings per participant and averaged to reduce random measurement error.
- The follow-up leveraged a well-characterised pregnancy cohort with documented hypertensive disorders of pregnancy, enabling adjustment for relevant clinical history and key behavioural/sociodemographic confounders.
- Flexible modelling approaches (restricted cubic splines and piecewise regression) were used to evaluate potential non-linear exposure–response relationships rather than assuming linearity.
- Limitations include potential selection bias due to loss to follow-up/migration, possible exposure misclassification because single-time water sampling may not fully reflect longer-term sodium intake or all dietary sodium sources, lack of urinary sodium biomarkers and single-time-point BP measurements conducted during warmer months without temperature adjustment.

## Introduction

 Globally, salinity intrusion—the influx of seawater into freshwater wetlands, rivers and aquifers—affects 11 Asian mega-deltas and other large estuaries, contaminating drinking-water supplies in several coastal cities.^1 2^ The Intergovernmental Panel on Climate Change Sixth Assessment Report projects that many large river basins across East, Central, South and Southeast Asia will face increasing water stress by midcentury, with freshwater availability varying widely by basin and season.^3^ At the same time, sectoral water demand across Asia is expected to rise by approximately 30–40% by 2050 compared with 2010, amplifying water stress even where mean runoff does not decline.^3^ Major river basins likely to be affected include the Ganges, Brahmaputra, Indus and Mekong, which provide water for hundreds of millions of people.^4^ Access to safe drinking water is therefore fundamental to achieving the United Nations Sustainable Development Goals, which seek to promote good health, ensure food security and reduce poverty.

In Bangladesh, an estimated 20–35 million people currently reside in salinity-affected areas, where drinking-water sources regularly reach sodium levels above 600 mg/L.^5 6^ During the rainy season, people largely have access to abundant harvested rainwater to drink, and heavy precipitation helps dilute the salinity of local drinking-water ponds, which act as mini-storage reservoirs. However, conditions deteriorate in the dry season, compelling people to rely on groundwater or on surface-water sources that become progressively more saline due to evaporation losses.^1 7^

Epidemiological studies provide strong evidence of a link between dietary salt intake and raised blood pressure (BP) and related cardiovascular complications, as shown in both clinical trials and animal studies.^8 9^ The WHO estimates that each year 1.89 million deaths are attributable to excess sodium consumption.^10^ Despite rising exposure of coastal populations to sodium in water, the contribution of drinking-water sodium (distinct from dietary salt) to BP remains under-studied, especially in low- and middle-income countries (LMICs).

A small number of studies have linked drinking-water salinity to pre-eclampsia and hypertension, as well as infant mortality, intrauterine growth restriction and preterm birth.^11^,^16^ However, WHO states that there is insufficient evidence on adverse outcomes linked specifically to sodium intake through water and that the levels typically found in drinking water globally (generally below 20 mg/L) are of no health concern.^17 18^ The only current guidance is an organoleptic cut-off of 200 mg/L—the level at which salty taste becomes perceptible and unpalatable—rather than a health-based threshold.^19 20^ This evidence gap fuels debate over whether health-based sodium standards are needed in climate-exposed coastal settings.

In 2008, we observed an unusual rise in rates of pre-eclampsia and hypertension among pregnant women in Dacope, a rural coastal subdistrict of Bangladesh, compared with non-coastal areas.^21^ It was hypothesised that increased salinity (sodium levels) in drinking water could be a contributing factor. Consequently, between 2009 and 2011, we conducted a population-based case–control study in Dacope to investigate the association between high-sodium drinking water and the risk of pre-eclampsia and gestational hypertension among pregnant women in coastal Bangladesh. We found exceptionally high sodium levels in drinking-water sources (mean 516.6 mg/L, SD 524.2), and women who consumed tube-well water (groundwater) were at higher disease risk than rainwater users (p<0.001). Adjusted risks for pre-eclampsia and gestational hypertension increased significantly in a dose–response manner with increasing sodium concentrations, and rates were considerably higher in the dry season, when salinity levels in surface and groundwater are elevated compared with the rainy season.^22 23^

For the present study, nearly a decade later, we followed up the same cohort of women to assess long-term changes in drinking-water salinity and the broader cardiovascular implications beyond pregnancy. While previous research—including our own—focused primarily on maternal complications, the contribution of drinking-water sodium to BP remains contested in low- and middle-income settings. Our primary research question was: What is the association between drinking-water sodium concentration and BP among non-pregnant women living in a high-salinity, climate-vulnerable coastal setting? To answer this, we aimed to: (1) quantify dose–response relations between drinking-water sodium and BP, testing for non-linearity and potential thresholds; (2) assess temporal change in drinking-water salinity across local administrative units between 2009 and 2018; and (3) evaluate whether reductions in sodium exposure over time correspond to improvements in diastolic BP.

By re-examining this unique cohort in a climate-vulnerable setting, the study provides rare longitudinal data and captures how salinity trends have evolved over time and their continuing health relevance. In doing so, this updated analysis addresses a critical global evidence gap on the cardiovascular risks of drinking-water salinity in LMICs and directly informs debates on standards for sodium in drinking water, surveillance and climate-resilient water policy in coastal regions.

## Methods

We conducted a follow-up study between April and July 2018 among participants from a previous cohort (2009–2011) in Dacope Upazila, a rural coastal subdistrict in Khulna District, southwestern Bangladesh.^11 23^ Dacope comprises nine administrative unions and 97 villages, with 36 597 households and a population of approximately 159 369 (2022 census). The area is intersected by two major tidal rivers—the Passur and the Shibsa—with semidiurnal, fortnightly and seasonal fluctuations in water levels ranging from 2 to 4.5 m.^24^

### Study population, sampling and eligibility criteria

The original study enrolled pregnant women aged 13–45 years at ≥20 weeks’ gestation, residing in Dacope Upazila between October 2009 and April 2011. Cases of pre-eclampsia, eclampsia or gestational hypertension were identified through the Upazila Health Complex and community surveillance; normotensive controls were randomly selected from the same source population, with four controls per case. In total, 1208 women were enrolled, including 202 cases and 1006 controls.

For the 2018 follow-up, all women enrolled in the original study who were alive, non-pregnant and still residing in Dacope were eligible. Women were excluded if they had migrated out of the study area, could not be located, declined participation or were pregnant at the time of follow-up. Participants were traced using original study identification records and approached sequentially by trained data collectors at their recorded addresses; if a woman was not found, the next eligible participant was approached. Of the original 1208 participants, 900 were located. Approximately 18% had migrated and were lost to follow-up, reflecting the high mobility typical of coastal populations.^25^ A total of 740 women were re-enrolled and included in the present analysis.

### Data collection

In 2009–2011, data were collected through structured interviews covering sociodemographic characteristics (eg, age, education, marital status, occupation, income), lifestyle factors (eg, smoking, alcohol use, physical activity, diet), body mass index (BMI), BP, pregnancy history and outcomes, and drinking water sources. Sodium concentrations were measured in participants’ drinking water.

In 2018, these measurements were repeated after 9 years of follow-up. A modified questionnaire captured the same domains plus water-related interventions since 2009. Participants were asked about their drinking water sources across three reference periods: the dry season, the rainy season and the time of data collection (April to July 2018). BP was measured following the same protocol used in the original study (described below).

### Patient and public involvement

Patients or members of the public were not involved in the design, conduct, reporting or dissemination plans of this research.

Community members and local stakeholders were involved during establishment of the original cohort through community workshops (uthaan baithak) with women, local leaders, teachers, non-governmental organisations (NGOs) and Upazila Health Complex staff. These discussions identified concerns about drinking-water salinity, seasonal water insecurity and perceived health risks, and informed the research question, field approach and local recruitment. Participants were not involved in the analysis or interpretation. Findings will be shared with relevant community and health-sector stakeholders through future dissemination activities.

### Sodium measurements

In 2018, we repeated salinity measurements in individual drinking water sources, both at participants’ homes (typically from storage containers) and directly from the source—such as tubewells, ponds, rivers or rainwater collection systems—used for drinking. For the present analysis, we used sodium concentrations measured from: (1) household-stored drinking water, and (2) the corresponding source from which each participant reported collecting their water.

In addition, 30 community-level samples (≈3 per union) were randomly collected from commonly used ponds and tubewells to estimate spatial variation in sodium levels across the region. Sampling and analysis followed American Public Health Association (APHA) (1998) protocols. These data were used to develop a spatial map of Dacope showing average sodium concentrations in key water sources.

Salinity, expressed in parts per thousand (ppt), reflects the concentration of total dissolved salts in water and is closely related to sodium levels, since sodium is a major ionic component. Electrical conductivity (EC; μS/cm) was used as a proxy and converted to sodium (mg/L), with cross-validation using direct salinity readings. Cation and anion analyses were performed at the Atomic Energy Centre in Dhaka. Further methodological details are provided in online supplemental table A.

### Primary health outcome

The primary outcome was BP, measured in millimetres of mercury (mm Hg) using manual sphygmomanometers following a standardised protocol.^26^ At least three readings were taken per participant, 5 min apart, and averaged for analysis. Systolic and diastolic BP were analysed as continuous outcomes and as dichotomous indicators of elevated BP, defined as systolic BP >120 mm Hg and diastolic BP >80 mm Hg. These thresholds were used to capture early BP elevation in this relatively young, largely normotensive cohort, rather than to define clinical hypertension. This approach is broadly consistent with the 2017 American College of Cardiology/American Heart Association (ACC/AHA) guideline, which classifies BP levels of 120–129/<80 mm Hg as elevated BP and ≥130/80 mm Hg as hypertension. As a sensitivity analysis, we also applied an ACC/AHA hypertension definition based on measured BP in 2018, defined as systolic BP ≥130 mm Hg or diastolic BP ≥80 mm Hg. Antihypertensive medication use could not be included because these data were unavailable.

### Statistical analyses

We computed summary descriptive statistics for all continuous and categorical variables. Because no health-based guideline exists for sodium in drinking water, we used WHO’s 200 mg/L taste threshold as the reference category, consistent with prior environmental health research.^27^,^29^ To assess associations between sodium exposure and BP, we applied restricted cubic spline regression with knots at the 25th, 50th and 75th percentiles to capture non-linearity. We also fitted a piecewise linear model with a single change point and compared linear, spline and piecewise fits to test departures from linearity.

Water sources were categorised as: (1) rainwater (reference), (2) pond water, including unfiltered pond water and water treated with pond-sand filters (PSFs) and (3) tubewell water (groundwater). Linear regression models examined associations between sodium exposure and continuous systolic and diastolic BP, while logistic regression models examined associations with dichotomous indicators of elevated systolic and diastolic BP. Models were adjusted for age, socio-economic status (SES), use of added salt in cooking or at the table and history of (pre)eclampsia.^11^ SES was assessed using a household asset-based index based on housing quality, household assets and utilities, cultivatable land ownership, sanitation, toilet sharing and education. Principal component analysis was used to derive an SES score, which was categorised into tertiles for analysis. Sensitivity analyses of the main logistic models additionally adjusted for marital status and chewing tobacco use; cigarette smoking, alcohol use and physical activity were not modelled because they were rare, unreported or unavailable. Analyses were conducted in Stata V.14.0 (StataCorp, College Station, Texas, USA).

## Results

A total of 740 women were included in the current analyses. Of these, 56 had experienced (pre)eclampsia and/or gestational hypertension between 2009 and 2011. None of the participants were pregnant at the time of data collection in 2018. The mean age of participants in 2018 was 30.3 years. Baseline characteristics are presented in table 1.

**Table 1:** Sociodemographic and environmental characteristics of participants (N=740)

	N (%)
Age in years, mean, SD	30.3 (4.4)
Education, n, %	
No education	53 (7.16)
Up to primary school	204 (27.6)
Class 6–9	372 (50.2)
Secondary school certificate	65 (8.78)
Higher secondary school certificate	29 (3.92)
Graduate studies	11 (1.49)
Postgraduate studies	6 (0.81)
Profession, n, %	
Daily labourer	18 (2.43)
Farmer, n, %	2 (0.27)
Service, n, %	8 (1.08)
Business, n, %	5 (0.68)
Housewife, n, %	658 (88.9)
Others, n, %	49 (6.62)
Marital status, n, %	
Married	733 (99.1)
Divorced/separated	7 (0.95)
Income, n, %	
No income	658 (88.9)
0–3000 taka	41 (5.54)
>3000–5000 taka	22 (2.97)
>5000–10 000	12 (1.62)
>10 000 taka	7 (0.95)
Chewing tobacco use*, n, %	
No gul	711 (96.1)
5 years	13 (1.76)
>5–10 years	14 (1.89)
>10 years	2 (0.27)
Socio-economic status†	
Low	247 (33.4)
Middle	247 (33.4)
High	246 (33.2)
Pre-eclampsia/hypertension during previous pregnancy, n, %	
Yes	56 (7.60)
No	684 (92.4)
Blood pressure, mean, SD	
Mean systolic BP	106.3 (SD 12.4)
Mean diastolic BP	71.7 (SD 9.2)
Mean sodium measured in stored water at home (mg/L), mean, SD	341.3 (725.7)
Mean salinity measured in stored water at home (ppt), mean, SD	1.07 (SD 2.77)
Mean EC measured in stored water at home, (mS/cm), mean, SD	1.71 (SD 3.63)
Mean sodium measured directly from water source (mg/L), mean, SD	327.4 (461.4)
Mean salinity measured directly from water source (ppt), mean, SD	1.02 (SD 1.61)
Mean EC measured directly from water source (mS/cm), mean SD	1.63 (SD 2.31)

* Cigarette smoking and alcohol use were not tabulated because only one participant reported smoking and no participants reported alcohol consumption. Chewing tobacco use was included as the more relevant tobacco exposure in this population.

† Socio-economic status was derived from a household asset-based index using principal component analysis and categorised into tertiles within the 2018 analytical sample.

BP, blood pressure; EC, electrical conductivity; ppt, parts per thousand.

Table 1 summarises the sociodemographic and environmental characteristics of participants enrolled in the cohort study (N=740). The table summarises age, education level, occupation, history of pre-eclampsia or hypertension in prior pregnancies, and baseline BP measurements. It also reports average sodium concentrations, salinity (ppt) and EC of stored drinking water and direct water sources at baseline.

### Drinking water sources used throughout the year

Online supplemental table B summarises drinking-water sources in dry and rainy seasons, ownership, costs and interventions over 9 years. Among 740 women, 5% reported purchasing drinking water—typically from government-operated or NGO-operated tubewells or tanks—at a cost of up to £6.75 per month. Approximately 66% changed water sources between seasons, citing lack of storage (36%), need for better quality (11%), unavailability of rainwater (14%) and insufficient access to freshwater (11%).

Dry season: In 2018, 27% used tubewells as their primary source (38% in 2009), followed by pond water (26%), rainwater (18%), filtered water (17%) and multiple sources (11%). Ownership was mixed: 12% government, 15% NGO, 3% private companies, 20% private households and 50% community-owned. During this season, 51% reported extreme difficulty in collecting water; ∼30% reported 30–60 min per collection and 9% >1–2 hours.

Rainy season: The primary source was rainwater (77%), followed by tubewell (12%), filtered (2%), pond (2%) and multiple sources (7%). Only 2% of sources were government-owned, 2.3% NGO, 1% privately owned and 80% were participants’ own sources, reflecting widespread rainwater harvesting. Compared with the dry season, fewer women (31%) reported extreme difficulty. However, 43 women (6%) reported needing more than a day to collect water from another village, often staying overnight on boats or trollers.

**Figure 1:**
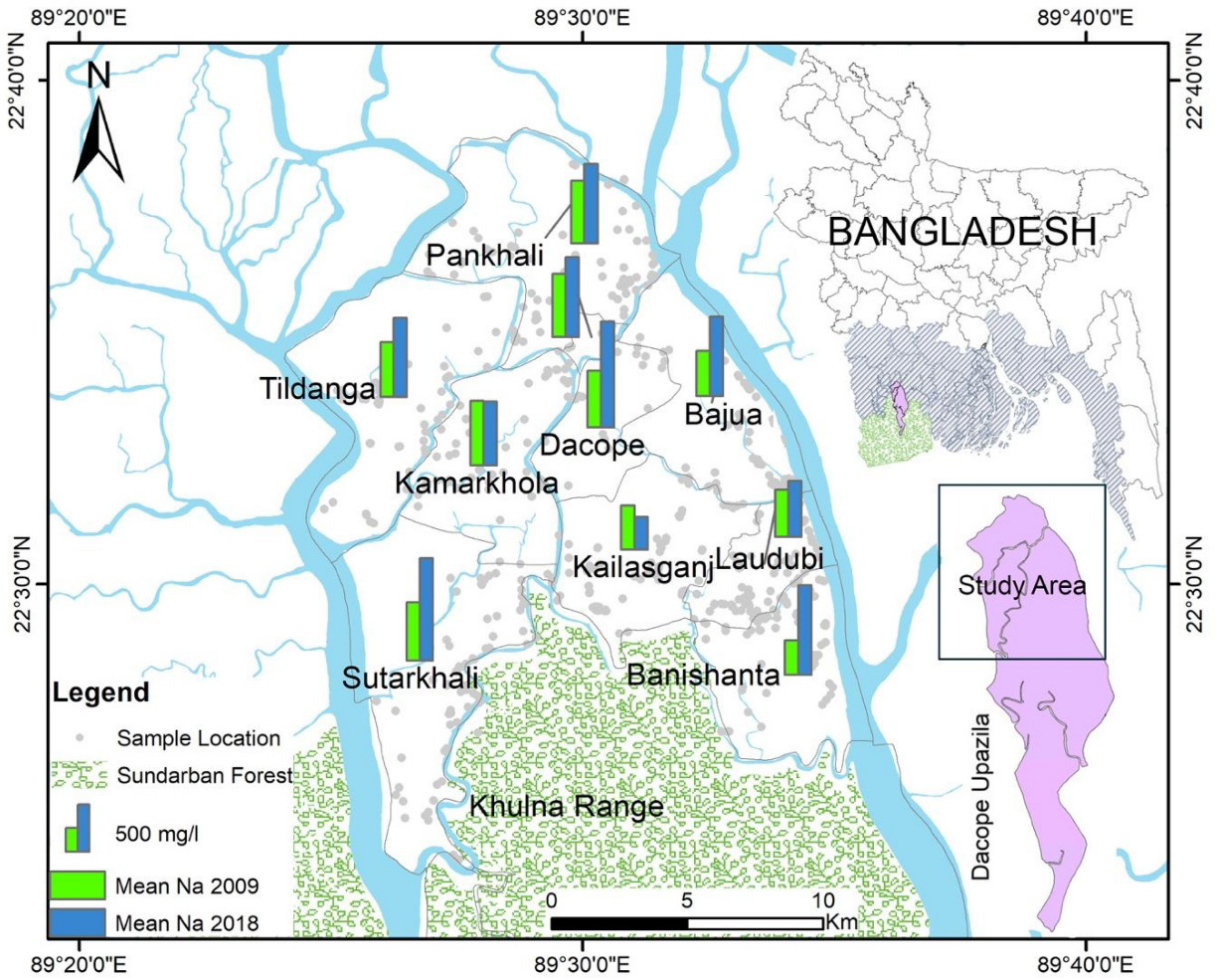
The figure displays the mean sodium levels in drinking water used by the participants of the study in 2009 and 2018 in the administrative units (unions) of Dacope subdistrict in Bangladesh. The map illustrates how sodium levels have increased in seven unions in Dacope over time.

Figure 1 presents a map of Dacope showing mean sodium concentrations in groundwater used for drinking between 2009 (mean=492.6 mg/L) and 2018 (mean=723.6 mg/L), based on measurements taken during April–June at the onset of the rainy season. In 2018, we resampled 2009 tubewells where possible; spatial coverage remained consistent across all nine unions. Sodium increased across seven of nine unions over 9 years (p=0.013), suggesting limited effectiveness of mitigation and/or worsening climate effects.

A consistent rise in salinity was observed in seven unions, with Dacope and Banishanta showing the largest increases. In Dacope, sodium rose from 537 mg/L (2009) to 1002 mg/L (2018), and in Banishanta from 326 mg/L to 847 mg/L (p<0.005). Kamarkhola and Kailasganj declined slightly, from 611 to 605 mg/L and 416 to 308 mg/L, respectively (p<0.005). Unions near tidal rivers—Bajua, Banishanta, Sutarkhali—had higher sodium; more central poldered unions (Kamarkhola, Kailasganj) tended to be lower.

### Sodium levels in household drinking water

**Figure 2:**
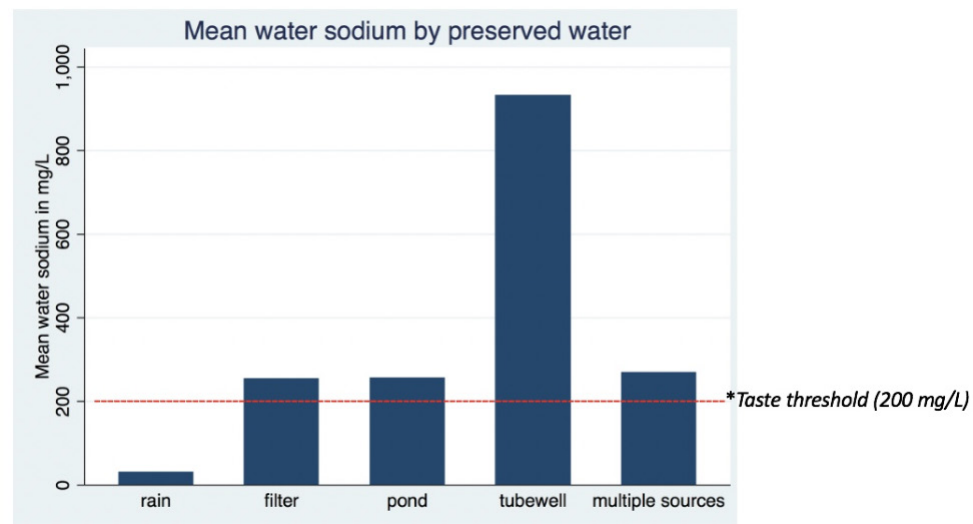
The figure shows the mean sodium levels in different drinking water sources used by the participants of the study. The red dashed line is not a health-based threshold but reflects the taste acceptability of water. The chart shows that all water sources, except rainwater, exceed this taste threshold, with tubewell water having the highest mean sodium concentrations. *The red dashed line represents the WHO taste threshold of 200 mg/L for sodium in drinking water, which is based on the point at which water is typically perceived as salty.

In 2018, mean sodium concentration in household drinking water—measured directly from containers across source types—was 341 mg/L. As shown in table 2, tubewell water had the highest sodium (mean 933 mg/L), with 77 participants consuming >1000 mg/L. This was followed by pond water (257 mg/L), filtered water (255 mg/L) and rainwater (30.0 mg/L). Participants using multiple sources reported an average of 269 mg/L. Sodium intake from tubewell water was 1.87 g/day, assuming 2 L/day consumption. Intake from rainwater was 0.06 g/day. Only rainwater met WHO’s 200 mg/L taste threshold; all other sources exceeded it (figure 2). Online supplemental table C presents sodium concentrations measured directly at the water source: tubewell water 917 mg/L, filtered 327 mg/L, pond 167 mg/L, other sources 61.7 mg/L, rainwater 30.6 mg/L.

**Table 2:** Sodium concentration (mg/L) measured in household drinking water

Water source	N=740 (%)	Mean sodium in mg/L	g/day with 2 L water intake	% of total recommended*
Rain	235 (31.8)	30.5	0.06	3
Filtered	75 (10.1)	255.2	0.51	26
Pond water	194 (26.2)	256.9	0.51	26
Multiple sources†	66 (8.92)	269.1	0.54	27
Tubewell	170 (22.9)	933.0	1.87	94

* Recommended daily limit is 2 g of sodium per day.

† A combination of different water sources.

Table 2 shows the mean sodium levels measured in different water sources used by the participants of the study, and the intake of sodium from each source, as a percentage of the total recommended daily limit of 2 g per day, as per the guidelines provided by the WHO (2025).

### Association of drinking water salinity with blood pressure

#### Mean sodium and blood pressure levels in 2009 vs 2018

**Figure 3:**
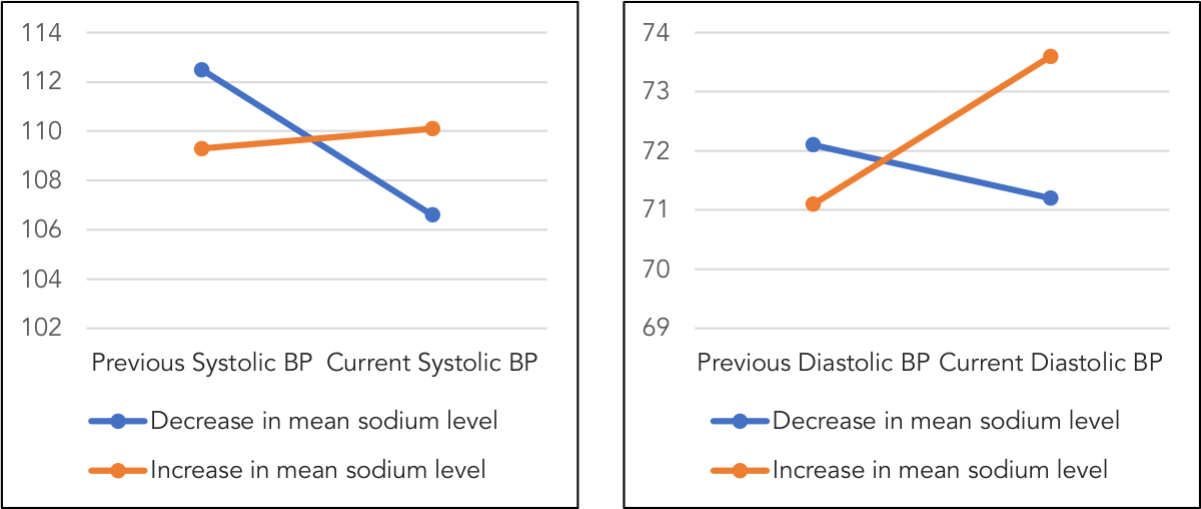
The figure shows the change in exposure levels between two time periods (2009 and 2018) and the corresponding change in BP between two groups. It illustrates how those with higher sodium levels in their drinking water in 2018, had higher mean BP levels. BP, blood pressure.

Among women without prior (pre)eclampsia/gestational hypertension (previous controls), mean systolic and diastolic BP in 2018 were 106.3 and 71.7 mm Hg—significantly higher than in 2009–2011 (103.0 and 67.0 mm Hg; p<0.001). Women with a prior history had higher BP in 2018 (112/75 mm Hg) than those without (105/71 mm Hg; p<0.001). Their mean drinking-water sodium was also higher (348 vs 310 mg/L; p=0.04). Women whose sodium exposure decreased between 2009 and 2018 showed BP reductions (systolic: 112.5–106.6 mm Hg, p=0.03; diastolic: 72.1–71.2 mm Hg, p=0.01; figure 3). Conversely, higher 2018 sodium corresponded to elevated BP (p-trend<0.001).

#### Association between water sodium levels and blood pressure in 2018

Using adjusted regression models, we examined the relationship between drinking water sodium concentrations and BP. In logistic regression models, each 100 mg/L increase in water sodium was significantly associated with higher odds of elevated diastolic BP (OR: 1.13; 95% CI 1.09 to 1.17), whereas no significant association was observed for elevated systolic BP (OR: 0.96; 95% CI 0.92 to 1.01).

As complementary analyses, we modelled systolic and diastolic BP as continuous outcomes. After adjustment for age, SES and added salt, each 100 mg/L increase in drinking water sodium was associated with a small, non-significant increase in systolic BP (β=0.16 mm Hg, 95% CI −0.05 to 0.37, p=0.133). In contrast, each 100 mg/L increase of sodium concentration was significantly associated with higher diastolic BP (β=0.31 mm Hg, 95% CI 0.15 to 0.46, p=0.001).

Using an ACC/AHA definition based on measured BP in 2018, 29.3% of participants met criteria for hypertension, defined as systolic BP ≥130 mm Hg or diastolic BP ≥80 mm Hg (217/740; 95% CI 26.1% to 32.7%). This was driven primarily by elevated diastolic BP: 28.9% of participants had diastolic BP ≥80 mm Hg (214/740), while 3.5% had systolic BP ≥130 mm Hg (26/740). Antihypertensive medication use was not included in this definition because medication data were unavailable.

In a sensitivity analysis using this ACC/AHA definition, each 100 mg/L increase in drinking-water sodium was associated with a modest increase in the odds of hypertension after adjustment for age, SES and added salt use (OR: 1.04; 95% CI 1.00 to 1.07; p=0.044).

**Figure 4:**
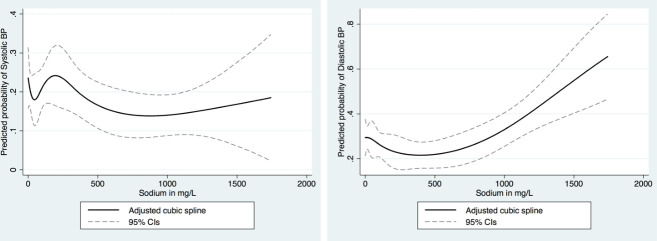
The figure shows the predicted probability of elevated systolic and diastolic BP across levels of sodium concentration (mg/L) in drinking water, modelled using restricted cubic splines. The black line is the adjusted fitted probability; grey dashed lines are 95% CIs. The spline model captures non-linear associations, with an apparent inflection point around 900–1000 mg/L, beyond which the probability of elevated BP increases. Estimates are adjusted for age, socio-economic status, added salt and history of (pre)eclampsia. Curves are shown over the observed sodium range; wider CIs at the extremes reflect sparser data. BP, blood pressure.

Figure 4 presents the dose–response relationships between sodium concentration in drinking water and the probability of elevated systolic and diastolic BP, modelled using restricted cubic splines with three knots. The models suggest non-linear trends, with the effects of sodium becoming more pronounced at concentrations exceeding approximately 1000 mg/L—indicating a potential threshold beyond which BP increases more sharply.

**Figure 5:**
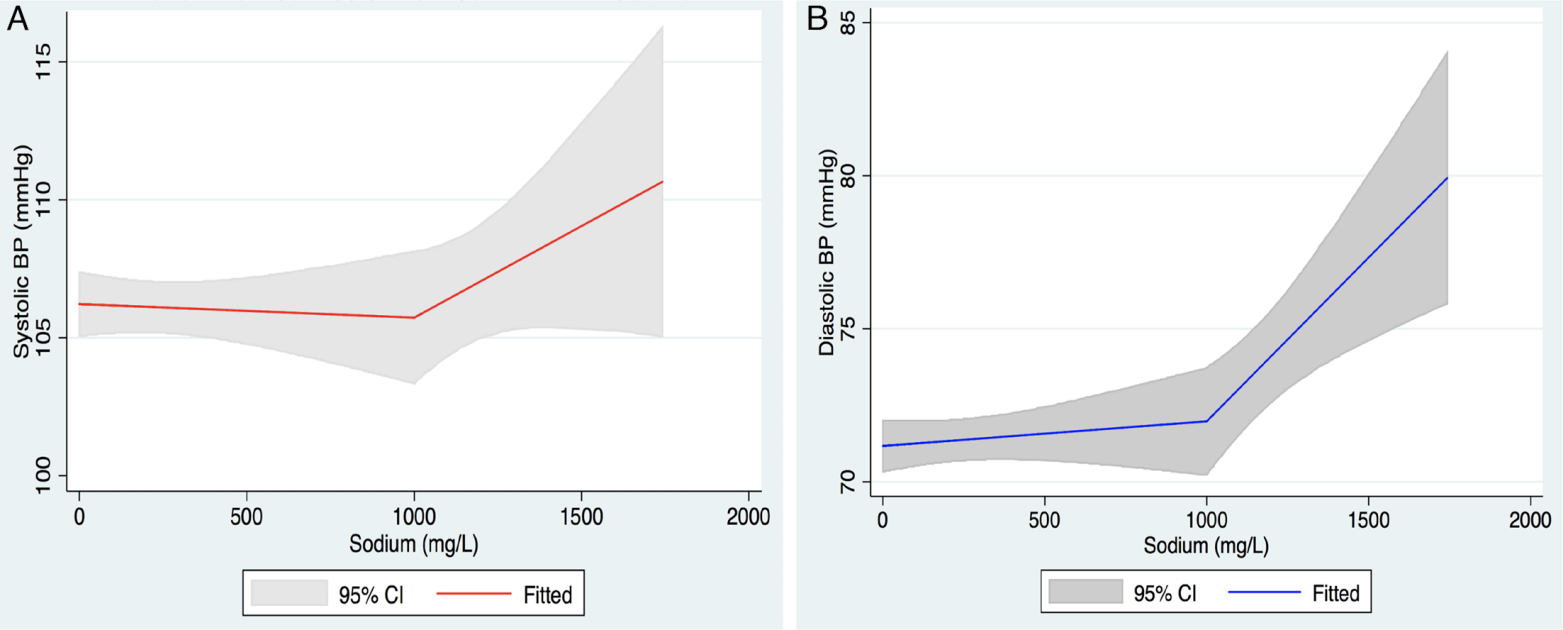
Piecewise linear regression models showing the fitted associations between sodium concentration (mg/L) in drinking water and (A) systolic BP and (B) diastolic BP. In both panels, the coloured line represents the fitted values, and the shaded area indicates the 95% CI. For systolic BP, a slight decline is observed below 1000 mg/L, with a steeper increase beyond this threshold. For diastolic BP, the model suggests a relatively flat or mildly increasing trend up to approximately 1000 mg/L of sodium, followed by a pronounced increase at higher concentrations. These patterns indicate a potential threshold effect in the relationship between sodium exposure from drinking water and BP, with higher values above 1000 mg/L associated with elevated BP risk. BP, blood pressure.

For diastolic BP, the adjusted curve is lowest around 500–700 mg/L of sodium, then rises steadily beyond 900–1000 mg/L. A piecewise linear model (figure 5) supports this pattern, showing a relatively flat or modestly increasing trend in diastolic BP up to around 1000 mg/L, followed by a noticeably sharper increase thereafter. Specifically, each 100 mg/L increase in sodium was associated with a 0.32 mm Hg rise in diastolic BP below 1000 mg/L (95% CI 0.13 to 0.51), and a larger increase of 0.56 mm Hg above 1000 mg/L (95% CI 0.15 to 0.85). While the spline model captures more nuanced curvature, both approaches consistently point to a sodium threshold near 1000 mg/L associated with elevated diastolic BP.

For systolic BP, the cubic spline model similarly indicates a non-linear association, with the curve relatively flat or slightly decreasing below 900–1000 mg/L, and rising gradually thereafter. These patterns suggest a more pronounced increase in risk above ∼1000 mg/L, although uncertainty increases at the distribution tails. The piecewise regression model (figure 5) shows a comparable trend: a slight, non-significant decrease in systolic BP below 1000 mg/L (−0.05 mm Hg per 100 mg/L; 95% CI −0.34 to 0.24; p=0.73) and a steeper, though not statistically significant, increase above this point (0.71 mm Hg per 100 mg/L; 95% CI −0.34 to 1.76; p=0.18). Despite wider CIs and lower precision in the systolic BP estimates, both models suggest a consistent inflection point near 1000 mg/L, beyond which systolic BP tends to increase.

**Figure 6:**
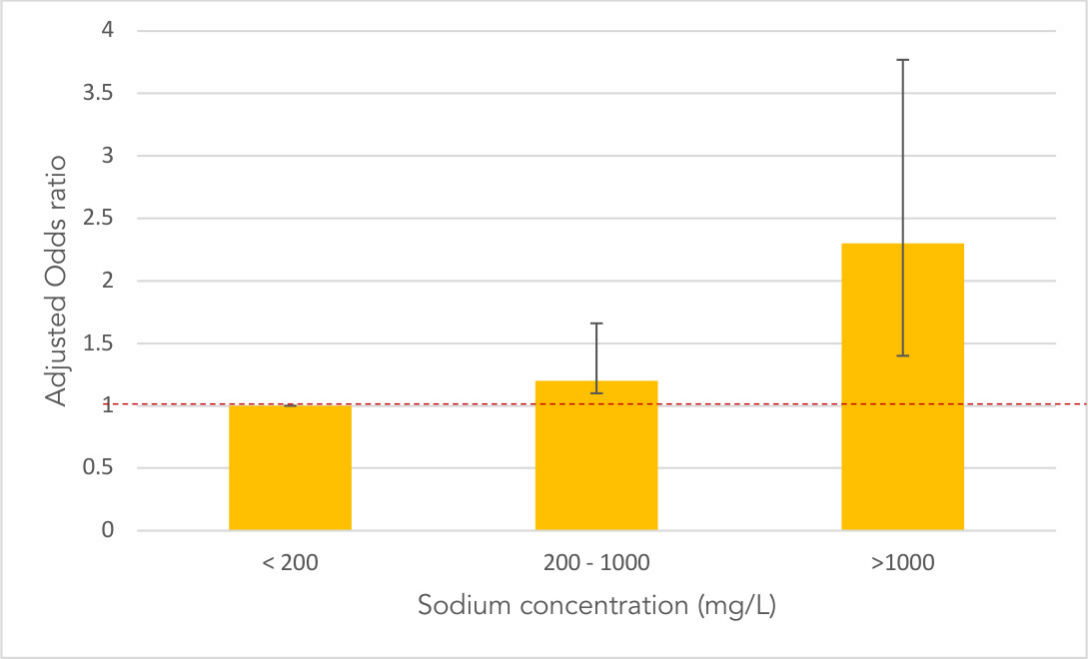
The figure shows the adjusted OR for diastolic BP by categories of sodium exposure, based on the WHO’s threshold for taste (200 mg/L) and the aforementioned threshold (1000 mg/L—found in the cubic spline models). The regression models were adjusted for age, history of (pre)eclampsia, socio-economic conditions and added salt. BP, blood pressure.

Table 3 categorises drinking water sodium concentrations into <200 mg/L, 200–1000 mg/L and >1000 mg/L (based on the WHO’s limit for taste threshold and the abovementioned threshold effect seen at 1000 mg/L). A progressive increase in mean systolic and diastolic BP was observed across these categories. In adjusted models, compared with those consuming water with <200 mg/L sodium (reference group), individuals in the 200–1000 mg/L group had a significantly higher risk of elevated diastolic BP (OR: 1.20; 95% CI 1.01 to 1.66; p<0.005) (figure 6). The association was even stronger for sodium levels >1000 mg/L (OR: 2.30; 95% CI 1.40 to 3.77; p=0.001), compared with<200 mg/L, suggesting a threshold or non-linear dose–response relationship. No statistically significant associations were observed for systolic BP across categories.

**Table 3:** Mean BP and adjusted odds of elevated BP by drinking water sodium category

Sodium (mg/L)	N	Mean SBP (mm Hg)	Adjusted OR* for SBP, 95% CI	P value	Mean DBP (mm Hg)	Adjusted OR* for DBP, 95% CI	P value
<200	279	106.0	1.00	–	71.1	1.00	–
200–1000	382	106.2	1.07 (0.70 to 1.65)	0.767	71.7	1.20 (1.01 to 1.66)	0.005
>1000	79	108.4	0.53 (0.30 to 1.10)	0.089	76.2	2.30 (1.40 to 3.77)	0.001

* Adjusted for age, SES, added salt and history of (pre)eclampsia

BP, blood pressure; DBP, diastolic blood pressure; SBP, systolic blood pressure; SES, socio-economic status.

Table 3 shows (1) the mean systolic and diastolic BP levels measured in participants categorised by three groups of sodium levels, and (2) the adjusted OR, 95% CIs and p value for elevated systolic and diastolic BP in relation to sodium levels. Regression models were adjusted for age, socio-economic conditions, added salt and history of (pre)eclampsia.

#### Association between water sources used in 2018 and blood pressure in 2018

Compared with rainwater users, pond/filtered, tubewell or multiple-source users had higher risk estimates for elevated BP, but associations were not statistically significant (online supplemental table D).

Results for the sodium-category models in table 3 were materially unchanged in sensitivity analyses additionally adjusting for marital status and chewing tobacco use. Cigarette smoking and alcohol consumption were not included as covariates because they were either extremely rare or not reported in this cohort, and physical activity data were not available for the 2018 follow-up.

#### Perceived taste and duration of exposure

During April–September (start of rainy season), 67% reported their water as ‘sweet’, 33% as ‘salty’. Perceived salty taste was associated with raised diastolic BP (OR 1.54; 95% CI 1.10 to 2.15; p=0.012), but not systolic BP (OR 0.62; 95% CI 0.41 to 0.94) (table 4). Duration of residence in Dacope was used as a proxy for long-term exposure. Longer residence was modestly but significantly associated with increased risk of both systolic and diastolic hypertension after adjustment, suggesting cumulative effects of high sodium exposure (table 5).

**Table 4:** Association between taste of water (sweet/salty) and SBP and DBP

Taste of water	N (%)	Mean sodium (mg/L)	Adjusted OR* for SBP, 95% CI	P value	Adjusted OR* for DBP, 95% CI	P value
Sweet	496 (67)	192.0	1	–	1	–
Salty	244 (33)	644.6	0.62 (0.41 to 0.94)	0.028	1.54 (1.10 to 2.15)	0.012

* Adjusted for age, SES, added salt and history of (pre)eclampsia

DBP, diastolic blood pressure; SBP, systolic blood pressure; SES, socio-economic status.

**Table 5:** Association between years in current house and systolic and diastolic BP

	Adjusted OR* for systolic BP	95% CIs	P value	Adjusted OR* for diastolic BP	95% CIs	P value
Number of years in current residence	1.04	1.02 to 1.08	0.002	1.10	1.02 to 1.14	0.017

* Adjusted for age, SES, added salt and history of (pre)eclampsia

BP, blood pressure; SES, socio-economic status.

Table 4 shows (1) the mean sodium levels categorised by perception of taste, and (2) the adjusted OR, 95% CIs and p value for systolic and diastolic BP in relation to the perception of taste. Regression models were adjusted for age, socio-economic conditions, added salt and history of (pre)eclampsia.

Table 5 shows the adjusted OR, 95% CIs and p value for systolic and diastolic BP in relation to the number of years spent in current residence. Regression models were adjusted for age, socio-economic conditions, added salt and history of (pre)eclampsia.

#### Interventions given between 2009 and 2018

Among 740 women, 296 (40%) changed primary drinking-water sources between 2009 and 2018 (online supplemental table B). Overall, 366 (49.5%) reported any water-quality intervention: government (3%), NGO (20%), private (<1%) or self-financed. Mean systolic BP did not differ significantly between women with versus without interventions (106.5 vs 106.0 mm Hg; p=0.53). Mean diastolic BP was higher among women reporting interventions (72.5 vs 70.8 mm Hg; p=0.01).

Interventions included household/community rainwater tanks (n=185; 27%), new tubewells (<1%), reverse osmosis (RO) plants (1%) and PSFs (3%). About 49% perceived their intervention as helpful, but numbers limited detailed subgroup analysis.

Mean sodium among those reporting any intervention was lower (247.5 mg/L) than among those without (432.9 mg/L; p<0.005). Despite lower sodium, BP was slightly higher in the intervention group (systolic BP 106.5; diastolic BP 72.5 vs systolic BP 106.0; diastolic BP 70.8), but exact timing of interventions during the 9-year period was not recorded, limiting interpretation.

## Discussion

### Salinity trends and blood pressure associations

This study examined changes in drinking-water salinity and its association with BP over time among women in coastal Bangladesh, using follow-up data from a cohort originally enrolled in 2009. We assessed sodium concentrations in current drinking water sources and explored seasonal variation in water sources, perceived salinity and water access patterns. Our findings offer new evidence on the health risks of environmental salinity exposure in a region increasingly affected by climate-related salinisation.

We found that sodium in drinking water constitutes a major source of daily sodium intake in this population. Assuming a conservative estimate of 2 L/day water intake, participants consumed up to 1.87 g/day of sodium from water alone—equivalent to 94% of the WHO’s recommended daily limit (2 g/day). Given that rural adults in southwest Bangladesh consume an average of 3.35 L/day,^29^ actual intake may be even higher. Approximately 22% of women consumed more than 1.80 g/day of sodium from drinking water, far surpassing the US Environmental Protection Agency’s recommended limit of 20 mg/L.^30^

This assumption is consistent with prior nutritional studies that use 2 L/day as a benchmark for adult fluid intake,^31 32^ though actual intake in hot environments may be substantially higher, increasing exposure to waterborne sodium. Unlike dietary sodium from food, which is often estimated through recalls or urinary biomarkers and may vary with preparation methods, sodium consumed through drinking water is almost completely absorbed in the gastrointestinal tract,^17^ making water an especially direct and measurable source of exposure.

This study provides evidence that elevated sodium concentrations in drinking water are significantly associated with increased diastolic BP among women in coastal Bangladesh. The non-linear nature of this association—confirmed through spline and piecewise regression models—suggests a possible threshold effect around 1000 mg/L, beyond which the impact on BP is more pronounced. The observed non-linear pattern, with a steeper increase in diastolic BP at higher sodium concentrations, may have important implications for future hypertension and cardiovascular risk in exposed populations. The categorisation of water sodium levels further supports this pattern, as the risk of elevated diastolic BP increased progressively across these categories.

We used elevated BP thresholds to capture early population-level BP shifts in this relatively young cohort, rather than to define clinical hypertension. This limits direct clinical interpretation but may be highly relevant for identifying emerging cardiovascular risk in populations chronically exposed to high-sodium drinking water.

Epidemiological evidence does not support a clearly defined biological threshold for sodium intake and BP; instead, most studies indicate a continuous dose–response relationship. The apparent threshold near 1000 mg/L should therefore be interpreted as a context-specific reference point for drinking-water sodium in this high-exposure setting, rather than a universal threshold for total salt intake. At this concentration, 2 L/day of drinking water would provide approximately 2 g/day sodium (5 g/day salt equivalent), before accounting for dietary sources.

For context, average salt intake in Bangladesh is 9 g/day nationally and 6–7 g/day in coastal populations.^33 34^ Notably, this estimate may understate intake in the most exposed coastal residents, as subgroup analyses showed higher odds of high salt consumption compared with hilly populations. This additional waterborne sodium may therefore substantially increase overall sodium exposure and contribute to the steeper rise in diastolic BP observed at higher concentrations.

Mean BP levels in 2018 were 3% higher for systolic and 6% higher for diastolic BP compared with baseline levels in 2009, likely reflecting the combined effects of ageing and cumulative exposure. Consistent with this, 29.3% of participants met an ACC/AHA hypertension definition in 2018 based on measured BP, although this estimate did not include antihypertensive medication use because these data were unavailable. Women with a history of (pre)eclampsia or gestational hypertension in 2009–2011 had higher mean BP in 2018, possibly reflecting both prior hypertensive pregnancy disorders and cumulative environmental exposure. This is consistent with prior studies showing long-term cardiovascular risks associated with (pre)eclampsia, including hypertension and stroke.^35^

We observed a temporal increase in sodium concentrations in drinking water between 2009 and 2018 in seven of nine unions, with the largest increases in Bajua, Sutarkhali, Banishanta and Dacope. These unions are located near tidal rivers and at lower elevations, where saline intrusion is more pronounced. This trend highlights the intensifying impacts of sea level rise, reduced freshwater flow and hydrological change in coastal Bangladesh.^7 36^ Identifying high-risk zones is critical for guiding health adaptation strategies. More broadly, our findings are relevant for other low-lying coastal and deltaic regions worldwide—including the Mekong, Nile and Mississippi deltas—where climate-driven salinisation of drinking water is also projected to threaten cardiovascular health.

### Seasonal variation, perception and access

Women in Dacope face seasonal water insecurity, particularly during the dry season, when reliance on high-sodium tubewell water is greatest. Over half of participants reported difficulty collecting water, and 1 in 10 spent over an hour doing so. These burdens are disproportionately borne by women, due to traditional gender roles in water collection. Despite rising salinity, fewer than half of participants reported receiving water-related interventions over the past 9 years, such as rainwater tanks, filters or RO units. However, due to missing information on the timing and consistency of these interventions, their effectiveness could not be fully assessed.

In hot tropical environments, sodium losses through sweating may be substantial. While elevated sodium in drinking water may increase BP among susceptible individuals, particularly those with salt sensitivity or elevated cardiovascular risk, it may also contribute to electrolyte replacement in the general population during chronic heat exposure, highlighting the need for context-specific interpretation.

Perceived salinity closely mirrored measured sodium levels: women who reported salty-tasting water had significantly higher sodium concentrations and higher diastolic BP. This suggests that perceived salinity may serve as a low-cost, community-level indicator of elevated exposure and potential health risk, and could be valuable for low-resource monitoring and awareness campaigns.

### Comparison with previous evidence

Our findings are consistent with a growing body of research linking high-sodium drinking water to elevated BP and adverse health outcomes. In our previous study in Dacope, we found that sodium concentrations were strongly associated with BP among non-pregnant adults^27^ and that high-sodium water during pregnancy increased the risk of (pre)eclampsia.^11 23^ A 2017 meta-analysis found positive associations between water sodium and BP in four of seven studies, particularly when sodium exceeded 150 mg/L.^37^ A 2023 review reported increased risks of cardiovascular, renal and maternal health outcomes in high-salinity regions of Bangladesh.^15^ More recent evidence also links chronic consumption of high-sodium water with elevated BP, cardiovascular disease, pregnancy complications and kidney disease.^16^ Additionally, emerging evidence suggests links between salinity exposure and developmental outcomes in children.^12 38^

Although some studies from high-income countries have reported inconsistent findings^39^,^41^—including trials showing cardiovascular benefits of sodium-rich mineral water^42 43^—these differ substantially in context. Involuntary, chronic exposure to saline water in low-resource coastal settings represents a distinct public health concern, particularly where communities have limited alternatives and weak infrastructure. In contrast, sodium levels in drinking water in high-income countries are typically too low to pose health risks, contributing to the perception that sodium in drinking water is negligible. These contextual differences highlight the importance of studying long-term, environmentally driven sodium exposure, especially in vulnerable populations.

This raises questions about current guidelines, which assume sodium intake from drinking water is negligible. While this may apply in high-income settings, it may not hold in salinity-affected coastal regions where water can substantially contribute to total intake. Our findings therefore support the need for context-specific evidence in developing drinking-water standards for sodium.

### Chronic exposure and salt sensitivity

We observed elevated BP among participants who had lived longer in the same residence, suggesting cumulative cardiovascular effects from chronic exposure to high-sodium water. This finding supports emerging evidence that prolonged exposure may increase salt sensitivity.^44^

Salt sensitivity—a heightened BP response to sodium intake—can arise from both genetic and environmental factors,^45^ including chronic stress and impaired kidney function.^46 47^ Animal studies indicate that early-life exposure to angiotensin antagonists increases susceptibility to salt-sensitive hypertension,^47^ while human studies link chronic psychosocial stress to heightened BP responses to sodium intake.^48 49^

In coastal Bangladesh, recurrent displacement, water scarcity and environmental hardship may exacerbate these risks—particularly among women. Early-life dehydration may also ‘program’ salt retention and elevate adult BP, consistent with the ‘thrifty phenotype’ hypothesis.^50^ This is especially concerning given the continued high prevalence of childhood diarrhoea in coastal regions of Bangladesh,^51 52^ which may compound cardiovascular risks associated with high-sodium water exposure.

Salt sensitivity is linked to increased cardiovascular disease and mortality, even among normotensive individuals,^53 54^ underscoring the potential long-term health consequences for populations exposed to saline drinking water.

### Sodium and isolated diastolic BP

Our analysis showed a significant positive association between drinking-water sodium and diastolic BP, but not systolic BP. Although sodium intake is often associated with increased BP through volume expansion and increased cardiac output, chronic sodium exposure may also influence peripheral vascular resistance, which more directly influences diastolic BP. Diastolic BP reflects arterial pressure during cardiac relaxation and is strongly influenced by vascular tone and systemic vascular resistance, whereas systolic BP is more closely related to stroke volume, cardiac output and arterial stiffness.^55^,^57^

This distinction may be particularly relevant in our relatively young study population (mean age 30.3 years), where arterial stiffness is lower and BP regulation may depend more strongly on vascular tone. Sodium-induced endothelial dysfunction, increased vascular reactivity and salt sensitivity may therefore preferentially affect diastolic rather than systolic pressure.^44 49 56 57^ Our findings are also consistent with previous evidence suggesting that high salinity drinking water may be more consistently associated with diastolic than systolic BP.^37^ Similar patterns were reported in hypertensive Chinese adults, where 24-hour urinary sodium excretion was significantly correlated with diastolic BP, but not systolic BP, after adjustment for age, sex, BMI, alcohol intake and season.^58^ Importantly, isolated diastolic BP is a modifiable risk factor for future cardiovascular events and progression to combined hypertension.^59^,^61^

The absence of a statistically significant association with systolic BP should be interpreted cautiously, however, as wider CIs and limited statistical power may have contributed to null findings. Further longitudinal studies with repeated BP measurements and urinary sodium biomarkers are needed to clarify the effects of drinking-water sodium on systolic BP over time.

### Strengths and limitations

Strengths of this study include its longitudinal design, use of standardised BP measurements and adjustment for key confounders including age, SES and cooking and table salt use. The integration of community perspectives on salinity perception and access to drinking water enhances contextual interpretation. This is among the few studies in LMIC settings to explore dose–response and threshold effects of environmental sodium exposure.

Limitations include a moderate sample size (n=740), loss to follow-up due to migration and reliance on self-reported histories. BP measurements were taken at a single time point during warmer months (April–July), when ambient temperature may influence BP levels, and detailed temperature data were unavailable for adjustment. However, measurements were conducted within a relatively narrow seasonal window, which may limit variability in temperature exposure within the study. Additionally, while we adjusted for known confounders, residual confounding cannot be ruled out. The absence of detailed data on dietary sodium intake and antihypertensive medication use may also have influenced the observed associations. We also did not have data on renal function or other cardiometabolic conditions that may influence sodium sensitivity and could therefore confound the observed associations. Urinary sodium biomarkers (eg, spot or 24-hour urinary sodium) were not collected, limiting our ability to assess total sodium exposure across all sources and potentially introducing exposure misclassification due to interindividual variability in water consumption and dietary sodium intake. Importantly, women lost to follow-up between 2009 and 2018 did not differ substantially in baseline sociodemographic or clinical characteristics from those retained in the study, suggesting that attrition is unlikely to have introduced major bias. Nonetheless, the consistency of results across models and exposure metrics strengthens confidence in the observed associations.

## Conclusions

Drinking-water salinity is an escalating, climate-amplified health hazard in coastal settings. Climate change is expected to exacerbate these trends, increasing risks of hypertension, cardiovascular disease, pregnancy complications and developmental impacts. In this cohort, higher drinking-water sodium was associated with elevated diastolic BP among non-pregnant women with a clear non-linear exposure–response. Within-person reductions in exposure corresponded to improvements in diastolic pressure. Average sodium concentrations were high and rose across most areas between 2009 and 2018, signalling a worsening exposure profile as sea-level rise accelerates saline intrusion.

In the absence of a WHO health-based guideline for sodium in drinking water, immediate policy and practice action are warranted: implementing routine salinity surveillance and rapidly expanding climate-resilient, low-sodium supplies (eg, rainwater harvesting, managed aquifer recharge, piped low-salinity sources), alongside locally tailored, community-informed options such as affordable desalination and seasonal storage. In parallel, targeted research should establish protective threshold levels to inform WHO standard-setting and evaluate the effectiveness, equity and scalability of mitigation strategies in coastal low- and middle-income settings. Embedding these measures within hypertension-prevention and climate-adaptation policies is essential to protect vulnerable communities now and over the coming decades.

## Supplementary material

10.1136/bmjopen-2026-116453 online supplemental file 1

## Data Availability

Data are available upon reasonable request.
