# Supplementary material for: Drinking water salinity and blood pressure in coastal Bangladesh: follow-up of a population-based cohort in a climate-vulnerable region
Source: BMJ Open. 2026 Jul 1;16(7):e116453. doi: 10.1136/bmjopen-2026-116453 (PMC13330963; doi:10.1136/bmjopen-2026-116453)
Supplement: online supplemental file 1 [file bmjopen-16-7-s001.docx]

# **Supplementary material**

**Table A.** **Instrumental process for major cation and anion detection**

| **Cation** | **Anion** |
| --- | --- |
| - **Analytical Column and Guard Column:** IonPac CS17, Ion Pac CG11 - **Eluent:** 6 mM MSA (Methanesulfonic acid) - **Flow rate:** 1 ml/min - **Operating Temperature:** 30°C - **Detection:** Suppressed conductivity - **Cation self-regeneration suppressor**: CSRS ULTRA II, 4mm - **Applied current:** 18 mA - **Injection volume:** 25 µl - **Storage solution:** Eluent (6 mM MSA) | - **Analytical Column and Guard Column:** Ion Pac AS11-HC and Ion Pac AG11-HC - **Eluent:** NaOH mM - **Flow Rate:** 1.5ml/min - **Anion Self-Generating Suppressor:** RFIC ASRC 300, 4mm - **Injection Volume:** 10µL - **Operating Temperature:** 30°C - **Pressure:** 1520(PSI) - **Detector:** DIONEX CONDUCTIVITY DETECTOR |
| **Operating Software:** Chromeleon, Version 6.80 | |

*Table A – Instrumental setup for ion chromatographic analysis of major cations and anions in drinking water samples. The table outlines key parameters including column specifications, eluent composition, flow rate, suppressor systems, and detection conditions. Cations were analyzed using a methanesulfonic acid (MSA) eluent with suppressed conductivity detection, while anions were detected using a sodium hydroxide eluent and a self-regenerating suppressor system. All measurements were performed at 30°C using the Chromeleon software (version 6.80).*

**Table B. Household Drinking Water Sources, Seasonal Variations, Access Challenges, and Perceptions in Coastal Bangladesh (N = 740)**

|  | **N (%)** |
| --- | --- |
| **Years in current residence, mean, s.d.** | 13.3 (5.46) |
| **Moved house in the past 8 years, n, %** |  |
| Yes | 133 (18.0) |
| No | 607 (82.0) |
| **Why did you move? n, %** |  |
| Economic reasons | 13 (1.76) |
| Environmental reasons | 96 (12.9) |
| Family | 21 (2.84) |
| Other reasons | 3 (0.41) |
| Not applicable | 607 (82.0) |
| **Main source of drinking water (used predominantly throughout the year), n, %** |  |
| Rainwater only | 161 (21.8) |
| Filtered water | 85 (11.5) |
| Pond water | 88 (11.9) |
| Tubewell water | 161 (21.8) |
| Others | 6 (0.81) |
| Multiple sources | 239 (32.3) |
| **Main source of drinking water source during the dry season, n, %** |  |
| Rainwater only | 130 (17.6) |
| Filter | 123 (16.6) |
| Pond | 192 (25.9) |
| Tubewell | 202 (27.3) |
| Others | 11 (1.5) |
| Multiple sources | 82 (11.1) |
| **Ownership of the source of water during dry season, n, %** |  |
| Government | 90 (12.2) |
| NGO | 113 (15.3) |
| Private company | 21 (2.84) |
| Self- owned | 151 (20.4) |
| Others | 334 (45.1) |
| I don't know | 31 (4.19) |
| **Individual or shared water in the dry season, n, %** |  |
| Family-based | 259 (35.0) |
| Community-based | 93 (12.6) |
| Village-based | 354 (47.9) |
| Union-based | 34 (4.59) |
| **Who takes care of the water in the dry season? n, %** |  |
| Self | 317 (42.8) |
| Family member | 32 (4.32) |
| Committee | 85 (11.5) |
| Upazilla Pouroshova members | 61 (8.24) |
| Community people | 139 (18.8) |
| Owners of the source | 87 (11.8) |
| NGO/other organizations | 11 (1.49) |
| Other | 8 (1.08) |
| **Difficulty for women to collect water in the dry season?** |  |
| Yes | 382(51.6) |
| No | 358 (48.4) |
| **Collection time in the dry season, n, %** |  |
| 0 - 10 minutes | 170 (22.9) |
| >10 - 30 minutes | 272 (36.8) |
| >30 minutes - 1 hour | 220 (29.7) |
| >1-2 hours | 64 (8.65) |
| >2 hours | 6 (0.81) |
| Don't know | 8 (1.08) |
| **Main source of drinking water source during the rainy season, n, %** |  |
| Rainwater only | 566 (76.5) |
| Filter | 18 (2.43) |
| Pond | 12 (1.62) |
| Tubewell | 89 (12.0) |
| Others | 5 (0.68) |
| Multiple sources | 50 (6.76) |
| **Ownership of the source of water during rainy season, n, %** |  |
| Government | 12 (1.62) |
| NGO | 17 (2.30) |
| Private company | 5 (0.68) |
| Self- owned | 593 (80.1) |
| Others | 110 (14.9) |
| I don't know | 3 (0.41) |
| **Individual or shared water in the rainy season, n, %** |  |
| Family-based | 653 (88.2) |
| Community-based | 30 (4.05) |
| Village-based | 48 (6.49) |
| Union-based | 9 (1.22) |
| **Who takes care of the water in the rainy season? n, %** |  |
| Self | 638 (86.2) |
| Family member | 10 (1.35) |
| Committee | 6 (0.81) |
| Upazilla Pouroshova members | 17 (2.30) |
| Community people | 35 (4.73) |
| Owners of the source | 28 (3.78) |
| NGO/other organizations | 6 (0.81) |
| Other |  |
| **Difficulty for women to collect water in the rainy season? n, %** |  |
| Yes | 233 (31.5) |
| No | 507 (68.5) |
| **Collection time in the rainy season, n, %** |  |
| 0 - 10 minutes | 297 (40.1) |
| >10 - 30 minutes | 197 (26.6) |
| >30 minutes - 1 hour | 124 (16.8) |
| >1 - 2 hours | 48 (6.49) |
| >2 - 5 hours | 28 (3.78) |
| More than 1 day | 43 (5.81) |
| Depends on the rain | 3 (0.41) |
| **Change of water source between seasons, n, %** |  |
| Yes | 489 (66.1) |
| No | 251(33.9) |
| **Why change water source between seasons? n, %** |  |
| No big storage tank/pot available | 267 (36.1) |
| To access better quality of water | 84 (11.4) |
| Rainwater collected not sufficient for the entire dry season | 33 (4.46) |
| Rainwater not available during the dry season | 74 (10.0) |
| Other reasons | 31 (4.19) |
| N/A | 251 (33.9) |
| **Change of water source in past 9 years, n, %** |  |
| Yes | 296 (40.0) |
| No | 444 (60.0) |
| **Were interventions given in the past 9 years, n, %** |  |
| Yes | 366 (49.5) |
| No | 374 (50.5) |
| **What was the intervention? n, %** |  |
| Family or community-based tank | 185 (25.0) |
| Deep tube-well | 1 (0.14) |
| PSF | 21 (2.84) |
| Filter water with pipe | 17 (2.30) |
| Filter by Reverse Osmosis | 8 (1.08) |
| Other | 76 (10.27) |
| Multiple interventions | 58 (7.84) |
| No intervention | 374 (50.6) |
| **Who provided the intervention? n, %** |  |
| Government | 21 (2.84) |
| NGO | 144 (19.5) |
| Private company | 2 (0.27) |
| Self | 156 (21.1) |
| I don’t know | 43 (5.81) |
| N/A | 374 (50.6) |
| **Type of intervention, n, %** |  |
| Family-based | 286 (38.7) |
| Community or village-based | 61 (8.24) |
| Union-based | 14 (1.89) |
| N/A | 374 (50.6) |
| Don’t know | 5 (38.65) |
| **How was it helpful? n, %** |  |
| Tastier/more pure water | 234 (31.6) |
| Able to access rainwater | 21 (2.84) |
| Able to access tubewell water | 30 (4.05) |
| Free from Disease | 9 (1.22) |
| Water from Somity | 39 (5.27) |
| Others | 26 (3.51) |
| N/A | 381 (51.5) |
| **How much money was needed for this intervention? n, %** |  |
| 0-500 taka | 38 (5.14) |
| >500-1000 taka | 2 (0.27) |
| >1000-5000 taka | 23 (3.11) |
| >5000-10000 taka | 76 (10.3) |
| >10000 taka | 42 (5.68) |
| I don't know | 185 (25.0 |
| N/A | 374 (50.6) |
| **Who pays for the intervention? n, %** |  |
| Self | 97 (13.1) |
| Family member | 79 (10.7) |
| Individuals/Person | 10 (1.35) |
| Government | 4 (0.54) |
| NGO/ other organization | 54 (7.30) |
| Don't know | 103 (13.9) |
| Upazila/Pouroshova | 7 (0.95) |
| Others | 12 (1.62) |
| N/A | 374 (50.6) |
| **Do you buy drinking water? n, %** |  |
| Yes | 38 (5.14) |
| No | 702 (94.9) |
| **How much money do you spend per month on buying water? n, %** |  |
| 0-50 taka | 7 (0.95) |
| >50-100 taka | 5 (0.68) |
| >100-500 taka | 21 (2.84) |
| >500-1000 taka | 5 (0.68) |
| N/A | 702 (94.9) |
| **Current taste of water, n, %** |  |
| Saline to some extent | 244 (32.9) |
| Sweet | 496 (67.1) |
| **Do you feel sick after drinking water? n, %** |  |
| Yes | 46 (6.22) |
| No | 694 (93.8) |
| **Purify water? n, %** |  |
| Yes | 133 (17.9) |
| No | 605 (81.8) |
| Don’t know | 2 (0.27) |
| **Do you perceive that there is a salinity problem in the region? n, %** |  |
| Yes | 417 (56.4) |
| No | 323 (43.7) |
| **What do you think the problems are? n, %** |  |
| Harmful for health | 52 (7.03) |
| Skin problems | 23 (3.11) |
| Physical problems | 46 (6.22) |
| Difficult to drink good quality water and carry out household work | 178 (24.1) |
| Not good for crops | 34 (4.59) |
| Not good for pregnant mothers | 36 (4.86) |
| Not good for any work | 22 (2.97) |
| Problem in accessing sweet (non-saline) water | 17 (2.30) |
| Others | 9 (1.22) |
| N/A | 323 (43.7) |
| **What initiatives have been taken? n, %** |  |
| Big tanks/pots provided | 458 (61.9) |
| Provided filters | 28 (3.78) |
| R.O plant | 13 (1.76) |
| Built tubewells | 53 (7.16) |
| Solo pump | 15 (2.03) |
| Card | 7 (0.95) |
| Arranged to preserve rainwater | 19 (2.57) |
| Arranged to provide sweet water | 23 (3.11) |
| Don't know | 124 (16.8) |

*Table B provides an overview of of water source use and access among surveyed households in coastal Bangladesh. The table includes data on seasonal variation in water sources, ownership, collection time, interventions, perceptions of salinity, and associated challenges—particularly during the dry season. These findings highlight the complexity of water management, gendered burdens in collection, and widespread concerns about the health impacts of saline water.*

**Table C. Sodium concentration (mg/L) in water sources (directly measured at source)**

| **Water source** | **N= 739 (%)** | **Mean sodium in mg/L)** | **g/day with 2L intake** | **% of total recommended*** |
| --- | --- | --- | --- | --- |
| Rain | 231 (31.2) | 30.6 | 0.06 | 3% |
| Other | 23 (3.11) | 61.7 | 0.12 | 6% |
| Pond | 267 (36.1) | 166.9 | 0.34 | 17% |
| Filtered | 35 (4.73) | 327.1 | 0.65 | 32% |
| Tubewell | 184 (24.9) | 917.3 | 1.83 | 92% |

*Table C shows the mean sodium levels measured directly from the water sources used by the participants of the study, and the intake of sodium from each source, as a percentage of the total recommended daily limit of 2 grams per day, as per the guidelines provided by the WHO (2025).*

**Table D. Association Between Water Source and Systolic and Diastolic BP**

| **Water Source** | **N (%)** | **Adjusted OR for SBP, 95% CI, p-value** | **Adjusted OR for DBP, 95% CI, p-value** |
| --- | --- | --- | --- |
| Rain | 235 (31.8) | 1.00 | 1.00 |
| Pond/filtered | 269 (36.4) | 1.13 (0.72 – 1.75), 0.611 | 1.03 (0.70 – 1.54), 0.150 |
| Tubewell | 170 (22.9) | 1.68 (0.40 – 1.19), 0.184 | 1.38 (0. .89 – 2.14), 0.50 |
| Multiple sources | 66 (8.92) | 1.24 (0.63 – 2.45), 0.533 | 1.10 (0.60 0 1.89), 0.000 |

*Table D shows the adjusted odds ratio, 95% confidence intervals and p-value for systolic and diastolic BP in relation to water sources. Regression models were adjusted for age, socioeconomic conditions, added salt and history of (pre)eclampsia*
