# Supplementary material for: Drinking water salinity and blood pressure in coastal Bangladesh: follow-up of a population-based cohort in a climate-vulnerable region
Source: BMJ Open. 2026 Jul 1;16(7):e116453. doi: 10.1136/bmjopen-2026-116453 (PMC13330963; doi:10.1136/bmjopen-2026-116453)
Supplement: Supplementary file 2 [file bmjopen-2026-116453.reviewer_comments.pdf]

PEER REVIEW HISTORY

BMJ Open publishes all reviews undertaken for accepted manuscripts. Reviewers are asked to complete a checklist review form and are provided with free text boxes to elaborate on their assessment. These free text comments are reproduced below.

ARTICLE DETAILS

Title (Provisional)

Drinking water salinity and blood pressure in coastal Bangladesh: follow-up of a population-based cohort in a climate-vulnerable region

Authors

Khan, Aneire Ehmar; Costopoulos, Eurydice; Mojumder, Sontosh Kumar; Shilpi, Asma Begum; Hoque, Mohammad A; Butler, Adrian; Millett, Christopher; Khan, Naila Zaman; Belesova, Kristine; Vineis, Paolo

---

VERSION 1 - REVIEW

**Reviewer**	**1**
**Name**	**Goto, Kenichi**
**Affiliation**	**Kyushu University, Health Sciences**
**Date**	**12-Feb-2026**
**COI**	**None**

This study is a follow-up to and an expansion of the authors' previous research. The findings regarding the significant relationship between drinking water salinity and diastolic blood pressure in coastal Bangladesh are compelling and hold great importance for public health. Overall, this is a well-structured and engaging manuscript. However, I have several comments that I believe would further strengthen the quality of the paper.

1. Please include information on marital status, income, smoking, alcohol use, physical activity, and diet in the Table 1 for baseline characteristics.

2. The results show that water sodium levels are associated with diastolic BP but not with systolic BP. Considering that the primary mechanism by which sodium intake increases blood pressure is through elevated cardiac output, leading to a rise in systolic BP—as the authors themselves noted—it is somewhat intriguing that an association was observed only with diastolic BP. Could you elaborate on the biological plausibility of the authors' claim “Dietary sodium has been shown to increase peripheral resistance and diastolic BP more than systolic BP, particularly in young population.”? A more detailed discussion of the underlying

mechanisms explaining why the association was observed exclusively for diastolic BP, and not systolic BP, would strengthen the manuscript.

3. High water sodium levels might be clustered among individuals with lower socioeconomic status (SES). Since low SES is a known risk factor for unhealthy behaviors (e.g., smoking, drinking, and physical inactivity) that contribute to blood pressure elevation, these variables may act as confounders. In the regression models, the authors adjusted for 'socioeconomic conditions.' Does this specifically refer to education level and occupation as listed in Table 1? I wonder whether the results presented in Table 3 would remain consistent if other factors, such as marital status, smoking, alcohol consumption, and physical inactivity, were also included as covariates in the analysis.

4. The observation of a clear threshold in the association between sodium concentration in drinking water and blood pressure levels is highly intriguing. Is the existence of such a threshold between salt intake and blood pressure elevation generally established in the existing literature? If so, what specific level of daily salt intake (in grams per day) is considered the threshold? I suggest that the authors expand their discussion on this point by incorporating data on the average daily salt intake in the participants' residential areas, which would provide a more comprehensive context for these findings.

**Reviewer**	**2**
**Name**	**Naser, Abu Mohd**
**Affiliation**	**The University of Memphis**
**Date**	**25-Mar-2026**
**COI**	**None**

---

1. The manuscript defines high systolic blood pressure as >120 mmHg and high diastolic blood pressure as >80 mmHg, treating these thresholds as dichotomous outcomes separately. These cut-points correspond more closely to “elevated blood pressure” rather than clinically defined hypertension and may have limited clinical interpretability when analyzed separately.

The authors may consider defining hypertension using established criteria, such as those from the American College of Cardiology / American Heart Association (e.g., ≥130/80 mmHg or antihypertensive medication use). In addition, modeling blood pressure as a continuous outcome (e.g., mean systolic and diastolic BP) would provide complementary insights into population-level shifts and may better capture the full exposure–response relationship with drinking water salinity.

2. It is unclear whether urinary sodium (e.g., spot or 24-hour urine sodium) was measured in 2018 to validate sodium exposure. If not, this should be more explicitly acknowledged as a limitation. Estimating sodium intake based on water sodium concentration and assumed

intake (e.g., 2 L/day with complete absorption) may introduce exposure misclassification, particularly given inter-individual variability in water consumption and dietary sodium intake. A biomarker such as 24-hour urinary sodium would provide a more integrated measure of total sodium exposure across all sources.

3. The observed increase in mean blood pressure between 2009–2011 and 2018 (e.g., systolic BP from 103.0 to 106.3 mmHg and diastolic BP from 67.0 to 71.7 mmHg) should be interpreted cautiously, as this may largely reflect aging of the cohort (approximately 9–10 years). To better contextualize the public health relevance, it would be helpful to report the prevalence of hypertension in 2018 based on standard criteria. This would provide a clearer picture of disease burden in the study population beyond changes in mean values.

4. Blood pressure measurements in 2018 were conducted between April and July, a period characterized by high ambient temperature and humidity in Bangladesh. Ambient temperature is known to influence blood pressure (typically inversely), and thus may act as a confounder. The authors are encouraged to consider adjusting for ambient temperature or to discuss this explicitly as a limitation, particularly when comparing blood pressure across time periods.

5. The observed association between drinking water sodium and diastolic blood pressure may be influenced by unmeasured comorbidities, such as impaired renal function or other cardiometabolic conditions, which could modify sodium sensitivity. The absence of such data should be acknowledged as a limitation.

6. Additionally, the broader interpretation could be more nuanced. In hot, tropical settings with high perspiration and limited access to cooling (e.g., air conditioning), sodium losses through sweating may be substantial. While elevated sodium in drinking water may increase blood pressure among susceptible individuals, its implications for the general population, particularly those without hypertension, may be helpful to replenish the lost sodium through sweating. A brief discussion of this physiological context would strengthen the interpretation.

VERSION 1 - AUTHOR RESPONSE

Reviewer 1 – Dr. Kenichi Goto

1. Add lifestyle and SES variables to Table 1

Comment: Please include information on marital status, income, smoking, alcohol use, physical activity, and diet in the Table 1 for baseline characteristics.

Response:
We thank the reviewer for this helpful suggestion. We have revised Table 1 to include additional available baseline characteristics, including marital status, household income/socioeconomic indicators, and tobacco use. In this study population, cigarette

smoking was extremely rare, with only one participant reporting smoking; therefore, we have reported chewing tobacco use instead, as this is a more relevant form of tobacco exposure among rural coastal women in Bangladesh. Alcohol use was not included in the table because no participants reported alcohol consumption. Physical activity was not included because comparable data were not available for the 2018 follow-up. Dietary information was collected in the original 2009–2011 study, but not in a comparable form at follow-up. Therefore, we did not include these historical dietary variables in Table 1, which is intended to describe the characteristics of the 2018 analytical sample. We have clarified these points in the Methods/Table 1 footnote as appropriate.

2: DBP vs SBP mechanism

Comment: The results show that water sodium levels are associated with diastolic BP but not with systolic BP. Considering that the primary mechanism by which sodium intake increases blood pressure is through elevated cardiac output, leading to a rise in systolic BP—as the authors themselves noted—it is somewhat intriguing that an association was observed only with diastolic BP. Could you elaborate on the biological plausibility of the authors' claim "Dietary sodium has been shown to increase peripheral resistance and diastolic BP more than systolic BP, particularly in young population."? A more detailed discussion of the underlying mechanisms explaining why the association was observed exclusively for diastolic BP, and not systolic BP, would strengthen the manuscript.

Response:

We thank the reviewer for this helpful comment. We agree that the stronger association with diastolic rather than systolic blood pressure warrants clarification. While sodium intake can increase blood pressure through volume expansion and increased cardiac output (affecting systolic BP), chronic sodium exposure may also increase peripheral vascular resistance through mechanisms such as endothelial dysfunction and salt sensitivity, which more directly influence diastolic BP.
These mechanisms may be particularly relevant in relatively young or normotensive populations, such as our cohort, where arterial stiffness is lower and BP regulation is more dependent on vascular tone. We have also noted that the absence of a statistically significant association with systolic BP may reflect limited statistical power rather than a true absence of effect. The Discussion has been revised accordingly.

3: Confounding by SES and behaviors

Comment: High water sodium levels might be clustered among individuals with lower socioeconomic status (SES). Since low SES is a known risk factor for unhealthy behaviors (e.g., smoking, drinking, and physical inactivity) that contribute to blood pressure elevation, these variables may act as confounders. In

the regression models, the authors adjusted for 'socioeconomic conditions.' Does this specifically refer to education level and occupation as listed in Table 1? I wonder whether the results presented in Table 3 would remain consistent if other factors, such as marital status, smoking, alcohol consumption, and physical inactivity, were also included as covariates in the analysis.

Response:

We thank the reviewer for this important comment. We have clarified in the Methods that socioeconomic status (SES) was assessed using a household asset-based index rather than education and occupation alone. This index was constructed from indicators of housing quality, household assets and utilities, income, land ownership, sanitation, toilet sharing, and education, to reflect material living conditions and resources in the rural coastal Bangladeshi context. Principal component analysis was used to derive the SES score, which was categorised into tertiles within the 2018 analytical sample.

We also conducted a sensitivity analysis additionally adjusting for marital status and tobacco exposure. Cigarette smoking was extremely rare in this cohort (one participant), and chewing tobacco use was therefore included as the relevant tobacco-related covariate. Alcohol consumption was not included as no participants reported alcohol use, and physical activity data were not available for the 2018 follow-up. The association between drinking-water sodium and elevated diastolic BP remained materially unchanged after these additional adjustments. These clarifications have been added to the Methods, Table 1 footnote, and Results sections.

4: Threshold evidence in literature

Comment: The observation of a clear threshold in the association between sodium concentration in drinking water and blood pressure levels is highly intriguing. Is the existence of such a threshold between salt intake and blood pressure elevation generally established in the existing literature? If so, what specific level of daily salt intake (in grams per day) is considered the threshold? I suggest that the authors expand their discussion on this point by incorporating data on the average daily salt intake in the participants' residential areas, which would provide a more comprehensive context for these findings.

Response:

We thank the reviewer for this important point. We agree that the threshold finding requires cautious interpretation. We have revised the Discussion to clarify that a universal threshold between total salt intake and blood pressure elevation is not established; rather, the wider literature generally supports a graded dose–response relationship between dietary sodium intake and blood pressure [1,2]. The WHO recommends limiting adult dietary sodium intake to <2 g/day, equivalent to <5 g/day

salt, but this is a recommended maximum population intake rather than a biological threshold for blood pressure elevation [3,4].

Importantly, there is also insufficient epidemiological evidence to define a health-based threshold specifically for sodium in drinking water. Although drinking-water salinity is an emerging threat affecting millions of people in vulnerable coastal and deltaic regions, relatively few studies have examined its cardiovascular effects, and previous studies have generally not attempted to examine non-linearity of the association and identify exposure thresholds even when sodium measurements were available. Therefore, the threshold observed in our study should be interpreted as an exploratory, data-driven threshold for drinking-water sodium in this high-exposure setting, rather than as an established guideline value.

In our study, the threshold near 1,000 mg/L refers specifically to sodium concentration in drinking water, not total dietary salt intake. Assuming 2 L/day water consumption, this corresponds to approximately 2 g/day sodium from drinking water alone, or about 5 g/day salt equivalent, before accounting for salt from food. We have added text to clarify this interpretation and to place our findings in the context of dietary salt intake in Bangladesh, where mean salt intake from food has been estimated at approximately 9 g/day in a nationally representative adult population, and 6.7 g/day among adults in climate-vulnerable coastal Bangladesh [5,6].

References

1. Huang L, Trieu K, Yoshimura S, et al. Effect of dose and duration of reduction in dietary sodium on blood pressure levels: systematic review and meta-analysis of randomised trials. *BMJ*. 2020;368:m315. doi:10.1136/bmj.m315.
2. Aburto NJ, Ziolkovska A, Hooper L, et al. Effect of lower sodium intake on health: systematic review and meta-analyses. *BMJ*. 2013;346:f1326. doi:10.1136/bmj.f1326.
3. World Health Organization. Sodium reduction. Geneva: World Health Organization; 2025.
4. World Health Organization. Reducing sodium intake to reduce blood pressure and risk of cardiovascular diseases in adults. WHO eLENA intervention guidance; 2023.
5. Monower MM, Biswas RK, Islam SMS, et al. Salt intake across the hypertension care cascade in the Bangladeshi adult population: a nationally representative cross-sectional study. *BMJ Open*. 2024;14:e081913. doi:10.1136/bmjopen-2023-081913.
6. Rasheed S, Jahan S, Sharmin T, et al. How much salt do adults consume in climate vulnerable coastal Bangladesh? *BMC Public Health*. 2014;14:584. doi:10.1186/1471-2458-14-584.

Reviewer 2 – Dr. Abu Mohd Naser

1: BP definition and continuous outcomes

Comment: The manuscript defines high systolic blood pressure as >120 mmHg and high diastolic blood pressure as >80 mmHg, treating these thresholds as dichotomous outcomes separately. These cut-points correspond more closely to “elevated blood pressure” rather than clinically defined hypertension and may

have limited clinical interpretability when analyzed separately. The authors may consider defining hypertension using established criteria, such as those from the American College of Cardiology / American Heart Association (e.g., ≥130/80 mmHg or antihypertensive medication use). In addition, modeling blood pressure as a continuous outcome (e.g., mean systolic and diastolic BP) would provide complementary insights into population-level shifts and may better capture the full exposure–response relationship with drinking water salinity.

Response:
We thank the reviewer for this helpful comment. We agree that our previous terminology could be interpreted as referring to clinically defined hypertension, whereas the thresholds used in the main analysis correspond more closely to elevated blood pressure. We have therefore revised the manuscript to refer to these outcomes as "elevated systolic BP" and "elevated diastolic BP," rather than hypertension.

We retained these lower cut-points for the primary analysis because the cohort was relatively young and largely normotensive, and our objective was to detect early population-level shifts in BP associated with drinking-water sodium exposure. In response to the reviewer's suggestion, we also conducted additional analyses modelling systolic and diastolic BP as continuous outcomes. These analyses showed a similar pattern to the main findings, with stronger evidence for an association between drinking-water sodium and diastolic BP than systolic BP. After adjustment for age, socioeconomic status, and added salt use, each 100 mg/L increase in drinking-water sodium was associated with a 0.31 mmHg higher diastolic BP (95% CI: 0.15 to 0.46; $p<0.001$). The corresponding association with systolic BP was smaller and not statistically significant ($\beta=0.16$ mmHg; 95% CI: −0.05 to 0.37; $p=0.133$).

We also conducted a sensitivity analysis using an ACC/AHA definition of hypertension, defined as systolic BP ≥130 mmHg or diastolic BP ≥80 mmHg based on measured BP in 2018. Antihypertensive medication use was not included because medication data were unavailable. In this analysis, each 100 mg/L increase in drinking-water sodium was associated with a modest increase in the odds of hypertension after adjustment for age, socioeconomic status, and added salt use (OR: 1.04; 95% CI: 1.00 to 1.07; $p=0.044$). This sensitivity analysis has been added to the Results, and the limitation regarding medication data has been clarified.

We have clarified in the Methods and Results that the primary outcomes represent elevated BP rather than clinically defined hypertension, and that the sensitivity analysis used an ACC/AHA threshold (≥130/80 mmHg).

2: Lack of urinary sodium

Comment: It is unclear whether urinary sodium (e.g., spot or 24-hour urine sodium) was measured in 2018 to validate sodium exposure. If not, this should be more

explicitly acknowledged as a limitation. Estimating sodium intake based on water sodium concentration and assumed intake (e.g., 2 L/day with complete absorption) may introduce exposure misclassification, particularly given inter-individual variability in water consumption and dietary sodium intake. A biomarker such as 24-hour urinary sodium would provide a more integrated measure of total sodium exposure across all sources.

Response:

Urinary sodium (either spot or 24-hour) was not measured in the 2018 data collection. We agree that this is a limitation, as our exposure assessment relies on estimated sodium intake from drinking water concentrations combined with assumed consumption levels, which may introduce exposure misclassification due to inter-individual variability in water intake and other dietary sodium sources. We have now explicitly acknowledged this limitation in the Discussion and highlighted that the absence of urinary sodium biomarkers (e.g., spot or 24-hour urinary sodium) limits our ability to capture total sodium exposure across all sources. We also note that future studies incorporating urinary sodium measurements would provide a more comprehensive assessment of sodium exposure

3: Aging of cohort

Comment: The observed increase in mean blood pressure between 2009–2011 and 2018 (e.g., systolic BP from 103.0 to 106.3 mmHg and diastolic BP from 67.0 to 71.7 mmHg) should be interpreted cautiously, as this may largely reflect aging of the cohort (approximately 9–10 years). To better contextualize the public health relevance, it would be helpful to report the prevalence of hypertension in 2018 based on standard criteria. This would provide a clearer picture of disease burden in the study population beyond changes in mean values.

Response:

We agree that the observed increase in mean BP between 2009–2011 and 2018 should be interpreted cautiously, as it may partly reflect aging of the cohort over approximately 9–10 years. To better contextualize the public health relevance of the findings, we have added the prevalence of hypertension in 2018 using an ACC/AHA definition based on measured BP. In 2018, 29.3% of participants met this definition, defined as systolic BP ≥130 mmHg or diastolic BP ≥80 mmHg (217/740; 95% CI: 26.1% to 32.7%). This was driven primarily by participants meeting the diastolic BP criterion (≥80 mmHg), whereas relatively few participants met the systolic BP criterion (≥130 mmHg). Antihypertensive medication use was not included because medication data were unavailable, and this has been noted as a limitation. We have added these results to the Results section and clarified the interpretation of changes in mean BP over time.

4: Ambient temperature

Comment: Blood pressure measurements in 2018 were conducted between April and July, a period characterized by high ambient temperature and humidity in Bangladesh. Ambient temperature is known to influence blood pressure (typically inversely) and thus may act as a confounder. The authors are encouraged to consider adjusting for ambient temperature or to discuss this explicitly as a limitation, particularly when comparing blood pressure across time periods.

Response:

We agree that ambient temperature may influence BP and could contribute to residual confounding, particularly when comparing BP across time periods. We have now acknowledged this as a limitation in the manuscript and clarified that BP measurements were conducted during warmer months (April–July), when ambient temperature may affect BP levels. All BP measurements in 2018 were conducted within the same seasonal window (April–July), with monthly average temperature between 29°C and 30°C and humidity between 70% and 81% (https://www.timeanddate.com/weather/bangladesh/khulna/historic?month=7&year=2018), reducing the likelihood of substantial within-study confounding by temperature.

5: Comorbidities

Comment: The observed association between drinking water sodium and diastolic blood pressure may be influenced by unmeasured comorbidities, such as impaired renal function or other cardiometabolic conditions, which could modify sodium sensitivity. The absence of such data should be acknowledged as a limitation.

Response:
We agree that unmeasured comorbidities, including impaired renal function and other cardiometabolic conditions, may influence sodium sensitivity and could therefore affect the observed association between drinking-water sodium and blood pressure. We did not have data on renal function or other cardiometabolic conditions in this cohort, and we have now acknowledged this as a limitation in the manuscript.

6: Sweating and sodium loss context

Comment: Additionally, the broader interpretation could be more nuanced. In hot, tropical settings with high perspiration and limited access to cooling (e.g., air conditioning), sodium losses through sweating may be substantial. While elevated sodium in drinking water may increase blood pressure among susceptible individuals, its implications for the general population, particularly those without hypertension, may be helpful to replenish the lost sodium through sweating. A brief discussion of this physiological context would strengthen the interpretation.

Response:

We appreciate this important contextual point and have added a brief discussion acknowledging that, in hot tropical environments, sodium losses through sweating may be substantial. While elevated sodium in drinking water may increase BP among susceptible individuals, particularly those with hypertension or salt sensitivity, it may also contribute to electrolyte replacement in the general population during chronic heat exposure. This nuance has been incorporated to provide a more balanced interpretation of our findings.

VERSION 2 - REVIEW

**Reviewer**	**1**
**Name**	**Goto, Kenichi**
**Affiliation**	**Kyushu University, Health Sciences**
**Date**	**05-Jun-2026**
**COI**	

The authors have addressed all the comments raised by the reviewer.
